# High-Precision AI-Enabled Flood Prediction Integrating Local Sensor Data and 3rd Party Weather Forecast

**DOI:** 10.3390/s23063065

**Published:** 2023-03-13

**Authors:** Qinghua Wang, Walid Abdelrahman

**Affiliations:** 1 Department of Computer Science, Faculty of Natural Sciences, Kristianstad University, SE-29188 Kristianstad, Sweden; 2 Department of Computer and Systems Sciences, Stockholm University, SE-16407 Kista, Sweden

**Keywords:** AI, artificial neural network, flood prediction, IoT, machine learning, time series data, weather forecast

## Abstract

Flooding risk is a threat to many sea-level cities and residential areas in the world. In the city Kristianstad in southern Sweden, a large number of sensors of different types have been deployed to monitor rain and other weather conditions, water levels at sea and lakes, ground water levels, and water flows in the city’s storm-water and sewage systems. All the sensors are enabled by battery and wireless communication, and allow real-time data to be transferred and visualized on a cloud-based Internet of Things (IoT) portal. To better enable the system with capacity of foreseeing upcoming flooding threats and to allow early response from decision-makers, it is desired to build a real-time flood forecast system by utilizing the massive sensor data collected at the IoT portal and data from 3rd party weather forecast service. In this article, we have developed a smart flood forecast system using machine learning and artificial neural networks. The developed forecast system has successfully integrated data from multiple sources and can make accurate flood forecast at distributed locations for the coming days. After being successfully implemented as software product and integrated with the city’s IoT portal, our developed flood forecast system has significantly extended the basic monitoring functions of the city’s IoT infrastructure. This article presents the context of this work, the challenges that have been encountered during our development, our solutions and performance evaluation results. To the best of our knowledge, this is the first large-scale IoT-based real-time flood forecast system that has been enabled by artificial intelligence (AI) and deployed in real world.

## 1. Introduction

Today, natural disasters such as flooding have a major impact on human beings and infrastructure. The cost of these disasters has increased a lot recently not only in an economic way but also the cost to the environment and the life of human beings. The increased cost is based on a few different parameters, and some of them may be the population growth and the change in land use patterns [1]. Water-related risks such as flooding since the year 2000 increased by 134 percent compared to the two earlier decades [2]. A great example of a change in land use pattern is the city of Kristianstad, which is in Scania County, Sweden. The location where Kristianstad was placed, was first a bay, and it was founded by the old king Christian IV year 1614. First, the city worked as a fortress where the water that flowed around worked as a protection for the city. Around the year 1860, a rebuild took place, which meant that a bay with water dried to create more space for cultivation. The dried area is placed below the sea level, and another part worth highlighting is that Sweden’s lowest ground point, also placed in Kristianstad which is located 2.41 meters below sea level [3]. Benefiting from AI and machine learning algorithms, more computing power, and the increased amount of data that are accessible, we can today make data analysis which benefit many aspects in our lives. This increasing trend has also influenced flood management, particularly in areas such as the development of flood prevention systems [4]. The potential and strengths of AI and machine learning are worth exploring and applying in areas of flood prevention.

The water and sewage system in Kristianstad municipality is under a digitization phase. By placing around 550 (which was the number according to our project plan but this number has been increased to 650 at the time of writing) sensors where the tasks of the sensors are to gather information about weather (including temperature, air pressure, and rain intensity), ground water level, and water flow information from the different water channels and pipes, collected data will then be sent to a cloud-based IoT portal for storage, processing and visualization. Through this digitization effort, the aim and goal are to make it possible to discover and prevent different errors and flood risks in the different areas of the municipality [5]. The raw data collected by the sensors however only tell the current and the history observations, but not the future observations. It requires advanced data analysis to learn data patterns, the relationship between history and future data, and the relationship between sensor readings and flooding risk. The earlier works on introducing the IoT project in Kristianstad and the preliminary machine learning results can be found in [6,7,8]. More specifically, correlations were identified between water flow data in the sewage water pipes under different time periods, and correlations were also found among flow data, ground water data and precipitation data. Therefore, a multi-linear regression model was built to predict future water flows in the sewage pipes in [6]. In [7], a long short-term memory (LSTM) neural network model was proposed to forecast water levels for a small set of test data. In [8], multiple artificial neural network (ANN)-based and LSTM-based machine learning models were proposed to forecast water levels. In particular, there was a performance evaluation and comparison among the models that were only based on data input from local sensors, the models that were only based on 3rd party weather forecast data, and the models that took both local data and 3rd party weather forecast data as inputs. In this paper, we are going to present our latest experiences on analyzing data from this IoT-based flood monitoring system. Among the other things, we are going to present an ANN and LSTM-based machine learning model that integrates data inputs from both local sensors and 3rd party weather forecast information. The developed system has been shown to be able to make multi-step forecast on future water levels at distributed locations (including underground wells, rivers and lakes) with high accuracy. To the best of our knowledge, this is the first large-scale IoT-based real-time flood forecast system that has been enabled by AI and deployed in real world for rapid flood disaster response. This article is an extended version of our earlier work [8].

In the following, we present related works in Section 2. We describe the two data sources used in this work in Section 3. How we have handled the difficulties in raw data are presented in Section 4. We have developed a machine learning model that can make multi-step forecast integrating data from multiple sources, and this model is presented in Section 5. The results are given in Section 6 where we also discuss model performances. Our reflections and potential future work are discussed in Section 7. Finally, we draw our conclusions in Section 8.

## 2. Related Works

Traditionally, storm water and flooding risk are analyzed using hydrology-hydraulic water flow simulation modelling tools like SWMM [9]. For example, a study was conducted in northern Stockholm in Sweden with the goal of exploring the effects on the drainage system from urbanization, city development, and potential increases in rain levels due to climate change. The research was mainly conducted using rain sensor data and processing the data in AutoCAD and SWMM to simulate the rainfall on the planned development of the additional drainage system as well as analyzing the typical way of handling rainwater in Sweden [10].

In the past decade, there have been a new trend on applying AI and machine learning algorithms on flood risk predictions around the world. Data can be collected via wireless sensor networks or via an IoT infrastrucutre. A hardware sensor prototype that can serve the purpose of flood monitoring is described in [11] and another one is in [12]. In [13], the authors not only develop a sensor prototype for flood monitoring but also manage to run a lightweight AI algorithm on their edge device which is a Raspberry Pi in this case. A systematic literature review on the applications of AI on disaster management (including flooding) at different phases has been made in [14], where an increasing number of publications on AI in disaster management has been observed in the past years. A real-time water level prediction model using artificial neural networks is introduced in [15] where it states that the recurrent neural network is capable of approximating nonlinear function and is efficient for water level prediction in Taiwan. Multilayer perceptron, a class of feedforward artificial neural network with back-propagation, is used for flood prediction in the Seybouse River Basin in Algeria country [16]. A short-term water and storm surge prediction model is proposed in [17] which introduced a neural network method in their work. They predict the surge levels after 5, 12, and 24 h with the help of hydrodynamic and meteorological data from the Tottori coast, Japan. Several different neural network models are evaluated against a real-world dataset in Pennsylvania but in a simulation environment where it is concluded that an LSTM-based approach has the best result on predicting downstream flow rate using upstream rainfall data [18].

There have also been research focusing on analyzing data collected from sewage water pipes for flooding forecast and other purposes. A previous study from Sweden explored if leaking drink water pipes could be a significant source for additional water in sewage pipes. The machine learning data analysis was made using simple linear regression and multiple linear regression analysis on data collected by the municipalities. The conclusion of the study found that there was a correlation between leakages in drink water pipes and additional water entering sewage treatment plants [19]. Hong Kong is a city that is located in subtropical region, and experiences heavy rainfalls and risks from the rise of the ocean. A study was conducted on potentially implementing a real-time monitoring system in drainage pipes using IoT-devices. The goal of the study was to create a smart drainage system using sensors and machine learning to be able to prevent potential flooding. Sensors collected water-level and water-flow data which was then processed using artificial neural networks [20]. A research comparing different AI-methods for analyzing inflow and infiltration in sewer sub-catchments was released in [21]. The aim of the research was to develop an AI-based system to analyze real-time inflow and infiltration data.

## 3. Data Collection

### 3.1. Sensor Deployment in Kristianstad

**Figure 1:**
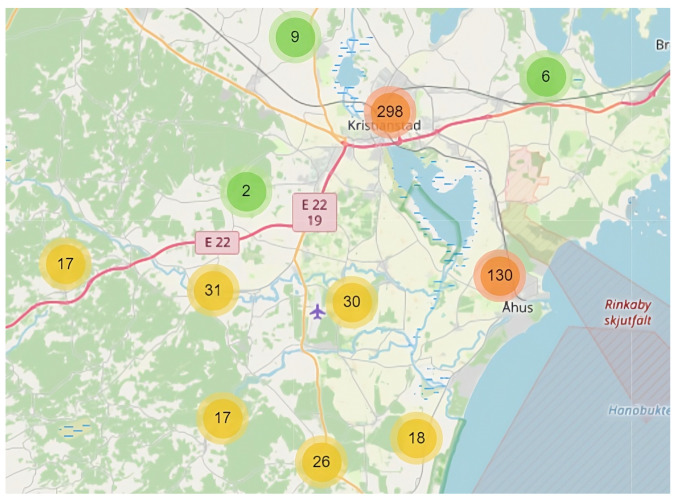
A graph that shows how sensors are deployed in Kristianstad. The graph is taken from the IoT portal of Kristianstad Municipality. Permission to publish is acquired from Kristianstad Municipality.

There have been around 550 sensors (and this number has been increased to 650 at latest) of different types deployed at and around the city Kristianstad. All sensors are enabled with wireless communication capability via the LoRaWAN [22] network provided by a local service provider. All sensor data are collected and visualized via an IoT portal. The data could be accessed remotely using scripts to allow real time processing and data analysis. The sensor deployment graph captured from the IoT portal is shown in Figure 1 where it is clearly shown that the sensors are clustered by their physical locations. Most of the sensors have been deployed in the city center where there are most of the surface channels and underground drainage water systems. Sensors are also deployed on the villages and small towns out of the city center. In addition, there are sensors deployed along the river flows which might be located in wild area. The types of sensors that are deployed include: water level, ground water level, sea water level, rain intensity, and flow speed. Each sensor can actually measure multiple types of attributes including temperature, humidity, air pressure, flow speed, water level, etc.

If we use all parameters from all sensors as data input, there will be more than two thousand dimensions. We thus group sensors according to their physical locations. In this work, we have grouped sensors into ten different groups where sensors which are nearby to each other have been put into the same group. One of the groups is called Åhus group and Åhus is a big town outside of Kristianstad where many sensors have been deployed. Another group is called Degeberga group and Degeberga is a small village in the southern tip of Kristianstad.

### 3.2. Weather Forecast

**Figure 2:**
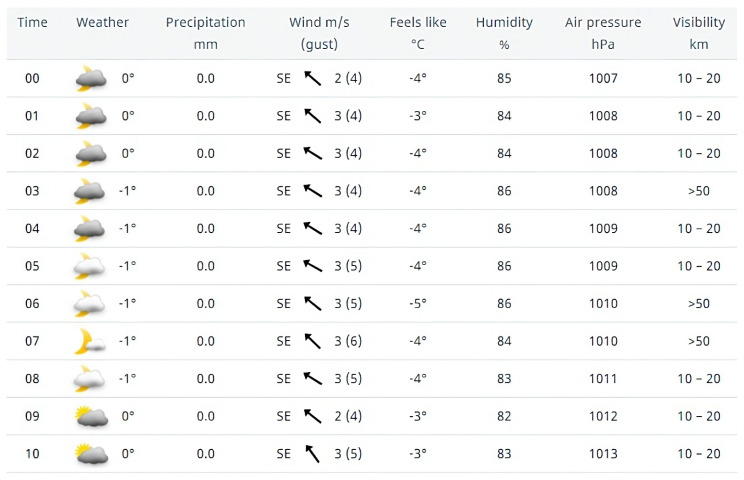
An example of weather forecast by SMHI (from SMHI website).

The weather forecast service in Sweden is provided by the Swedish Meteorological and Hydrological Institute (SMHI) [23]. SMHI provides 10-day weather forecast for the whole country. It is possible to download forecast data from SMHI database using longitude and latitude information. The weather forecast data are inferred from satellite data and local weather stations. The local weather stations in Kristianstad unfortunately only observe weather conditions twice per day. The nearest weather station that samples weather data once per hour is located in Hörby which is 40 km south of Kristianstad. The types of data included in the SMHI forecast are temperature, precipitation, wind, sunrise/sunset, etc., as well as a value on forecast certainty which falls into three categories: Certain, Fairly Certain and Uncertain. The SMHI forecast has a high accuracy in the coming 72 h, but worse accuracy in a longer period. For the short-term forecast within 72 h, the values are updated once per hour. For longer forecast, the values are updated three times per day or less. An example of weather forecast from SMHI is shown on Figure 2.

Though we can download future weather forecast information from SMHI at real time, we do not have access to the history weather forecast which is a paid service and presumably not available at real time either. If we are going to build a machine learning model using weather forecast information, the history data is required for model training and validation (and real-time data is required for updating model on the fly and for real-time prediction). By comparing the history data observed by SMHI’s local weather station at Kristianstad (which has a low resolution like 1–2 samples per day and contain only rain and temperature values) and the sensor data collected by Kristianstad Municipality (which has a high resolution with multiple samples per hour), it is found that the two sources of data agree with each other after we filter out errors and noise. We therefore propose to use the municipality’s sensor data to estimate SMHI’s history weather forecast.

**Figure 3:**
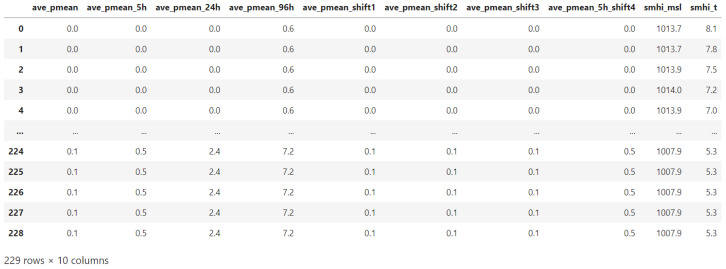
Derived weather parameters from SMHI. The same set of parameters are also generated from local IoT sensors as estimation of SMHI’s history forecast.

There are 19 weather parameters in the forecast data provided by SMHI, and the municipality’s sensors cannot observe all of them. But the important parameters like the rain can be observed by the municipality’s own sensors. In addition, many sensors also measure temperature and air pressure. We thus use a subset of SMHI weather parameters in this study, and this subset includes precipitation, temperature and air pressure. In addition, we have observed a delay of rain effect on the water levels, and have created derived parameters based on accumulated precipitation and time shifted precipitation. Our final data set include 10 parameters for future SMHI weather forecast and its estimated history data. Figure 3 shows a screenshot of the derived weather parameters which are going to represent the weather forecast values. In Figure 3, ave_pmean is the average precipitation in millimeter per hour, ave_pmean_5h is the accumulated average precipitation in the last five hours. ave_pmean_24h is the accumulated average precipitation in the last 24 h, ave_pmean_96h is the accumulated average precipitation in the last 96 h, ave_pmean_shift1 is the average precipitation with one hour delay, ave_pmean_shift2 is the average precipitation with two hours delay, ave_pmean_shift3 is the average precipitation with three hours delay, ave_pmean_5h_shift4 is the accumulated average precipitation in the last five hours with four hours delay, smhi_msl is the air pressure, and smhi_t is the temperature. In addition, each row in the dataset represents a period of one hour.

## 4. Data Pre-Processing

There are two data sources: one from local sensors, and one from weather forecast. The weather forecast data are actually predicted by SMHI’s own algorithms based on their own data and are therefore very well structured. For each attribute in the SMHI weather forecast, there is a value on every hourly time point in the first 72 h, and then a value every three hours, every six hours, or every 12 h by the end of the 10-th day in their forecast period. For the SMHI data, we do not need to do much pre-processing other than an upsampling to make sure there is a value on every hourly time point even after 72 h. There is, however, a necessity to frequently update the SMHI data because the forecast data is updated every hour by SMHI and the new forecast has taken the newest weather development into account.

The data from local sensors are actually not from one single place, but from 550 sensors of different types (and this number has been increased to 650 at latest). These sensors have been configured with different sampling frequencies, ranging from ten minutes to one hour. The sensor data are forwarded to an IoT portal via its nearest gateway, and data can be lost during transmission. Missing data can also be due to depleted battery, or errors and sometimes scheduled maintenance in the cloud server (which might happen once per month according to our experience). Besides the missing data problem, the data accuracy from local sensors appear to be also a problem. For example, there are offsets between actual measurements and the real values. The municipality makes calibration for each individual sensor before putting it into service. Errors like outliers cannot be calibrated by the municipality. We remove outliers in different ways. For temperature in the observation period, if the observed temperature is more than 40 Celsius degree or lower than −10 Celsius degree, we will reset them to 40 or −10. We do the same for air pressure if it is lower than 900 or more than 1100. The upper bounds and lower bounds are set according to our experience. For the rain sensors, we actually find out that many rain sensors do not report correct data by reporting no rain during rainy days (which can be due to missing data in a long period but can also be due to other reasons) or their reported data differ much from the observed data by SMHI (which has better accuracy). We therefore use statistical methods to fix these errors, by setting lower and upper bounds to respectively the 25th percentile and the 90th percentile. Whether it should be 25th percentile or 10th percentile for the lower bound can be discussed. Since we have observed there are non-working sensors with missing values and the missing values are often backward filled with 0 (and therefore below the lower bound), we use a slightly large number (i.e. 25th percentile) to exclude both the sensors with missing values and the sensors that report unreasonably small values. Because the strange values are very possibly wrong values and only the average value of all rain sensors are used in data analysis, their removal or replacement do not affect our results in a negative way.

After removal of outliers, we create a global parameter for temperature based on readings from all temperature sensors. We also create global parameters for air pressure and rain in similar ways. The global parameters take the average value of all sensor readings (after data pre-processing) and are more close to ground truth than sensor readings from individual sensors. We have made comparisons between the global parameters derived from local IoT sensors and the data from SMHI’s observation station at Kristianstad (which has a low temporal resolution but high accuracy). They coincide very well.

There are also other problems in the raw data which could cause problems in later machine learning. One problem is the constant data for some attributes in the observation period. The values of some attributes, like battery level and radio signal quality, change quite slowly over time and they do not contribute to the flooding prediction which is the purpose of our work. As a result, we identify and remove these constant or semi-constant variables during our data pre-processing. Another issue is that data from multiple sources are not aligned and they have different frequencies. This issue has been fixed via data re-sampling. There is also an issue of data dependency. The values of some attributes are actually calculated based on the values of other attributes by the end devices (i.e., the sensor board). For example, it is found that flow velocity and water depth measured by the same sensor are highly correlated. Most of the sensors provide information about temperature, humidity, and air pressure information. Though these sensors neither take samples at the same time, nor at the same location, these data are highly correlated. We therefore do not need so many measurements, and create a global parameter for each of these features instead. The removal of dependent data decreases the dimension of data inputs and reduces the complexity of our problem.

## 5. Methodology

This section presents our methodology on organizing the data and feeding them into the machine learning model. We also present the details of the proposed machine learning model and give a brief motivation behind the model development.

### 5.1. Time Series Data

We are dealing with time series data in this article. The sensor readings have timestamps. Forecast values from SMHI have timestamps. Our target is also to forecast future flooding risks (in terms of water levels) at specified future time stamps. In our data pre-processing, we have proposed to re-sample all data to one data point per hour. It is easier to talk about the discrete time concept time step instead of timestamp which could be continuous. In our case, one time step corresponds to one hour time interval.

**Figure 4:**
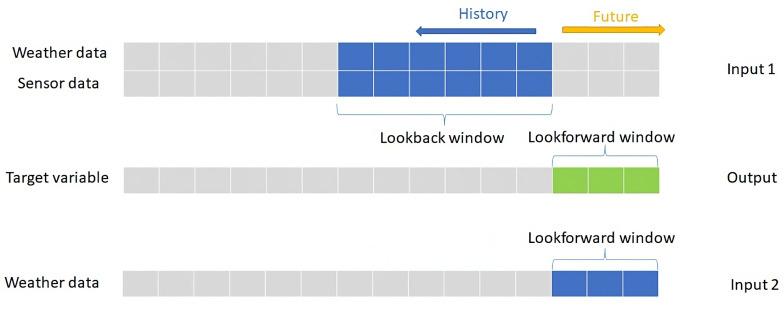
The relationship between input data and output data from time perspective.

We are going to use both history data and future weather forecast to predict flooding risks in our model. The amount of history data we are looking at is specified in a lookback window in terms of the number of time steps back in time from the current time of interest. The amount of future data we are aiming to predict is specified in a lookforward window in terms of the number of time steps ahead in time from the current time of interest. Figure 4 shows the lookback and the lookforward time windows, and how the input data and output data for the machine learning model (explained later) are related to each other from the time perspective. In this case, the window length for the future weather data is the same as the lookforward window. If we want to utilize the weather forecast to make flood prediction, we are basically also limited by the capacity of the 3rd party weather forecast service (including their accuracy and the number of days they can forecast). It is out of the scope of this article to explore the influence of weather forecast on the result. We do not have history weather forecast data, but we can estimate history weather data using local IoT sensor data as explained in Section 3. The estimated history weather data are used in together with the raw sensor data in the same lookback window on real-time model prediction, while the real-time forecasted weather from SMHI is used in the lookforward window. During model training phase, the estimated history weather data appear both in the lookback window and in the lookforward window.

### 5.2. Model Architecture

There are two data sources: local sensors, and SMHI forecast. Both data sources could provide some insights to the predictions of flooding risks. Flooding risks can be defined by water depths in the sea/river and underground wells, their duration and future trend, and the surrounding environments to the wells/rivers/sea. In this work, we focus on the prediction of future water depths in the locations where sensors have been deployed. In our previous paper [8], we have presented three categories of model architectures classified by the model inputs: 1. Local sensor data as input; 2. SMHI forecast data as input; 3. A combination of local sensor data and SMHI forecast data as inputs. In each category, we have presented several model architecture with different motivations. If the readers are interested, they can refer to our original paper for details. In this article, we have chosen to ignore all the models we have developed on the way but only focusing on the model that we have used in our final delivery.

**Figure 5:**
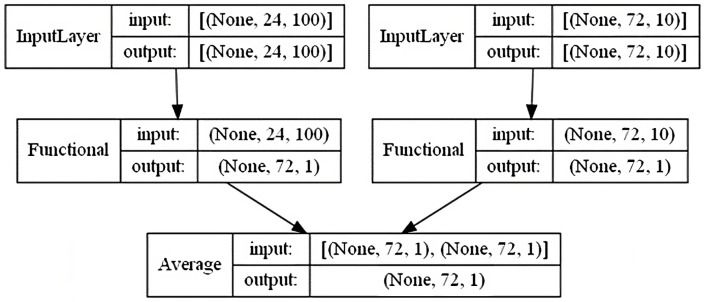
Model architecture: a model that considers both local sensor data and SMHI weather forecast. The functional units include multiple layers which are explained in Figure 6 and Figure 7.

The model architecture is shown in Figure 5. We have chosen Keras [24] Functional API to implement this graph topology. In comparison to a simple sequential model, Keras Functional API allows a user to build non-linear and complex model topology (e.g., with multiple inputs as in this case). In our model, there are two inputs and one output. The InputLayer on the left is for history data from local sensor. The InputLayer on the right is for weather forecast from SMHI. Input 1 has the shape (n_samples, n_steps_lookback, n_input_features) and represents the history data from local sensors and the history weather forecast (also estimated using local data), and input 2 has the shape (n_samples, n_steps_lookforward, n_smhi_features) and represents future weather data which can be downloaded at real time via a program API from the 3rd party SMHI [23]. The shape parameters, their definitions and their values are given in Table 1. These parameters are adjustable. At the end, the target value is estimated by taking an average from the outputs of the two functional units.

**Figure 6:**
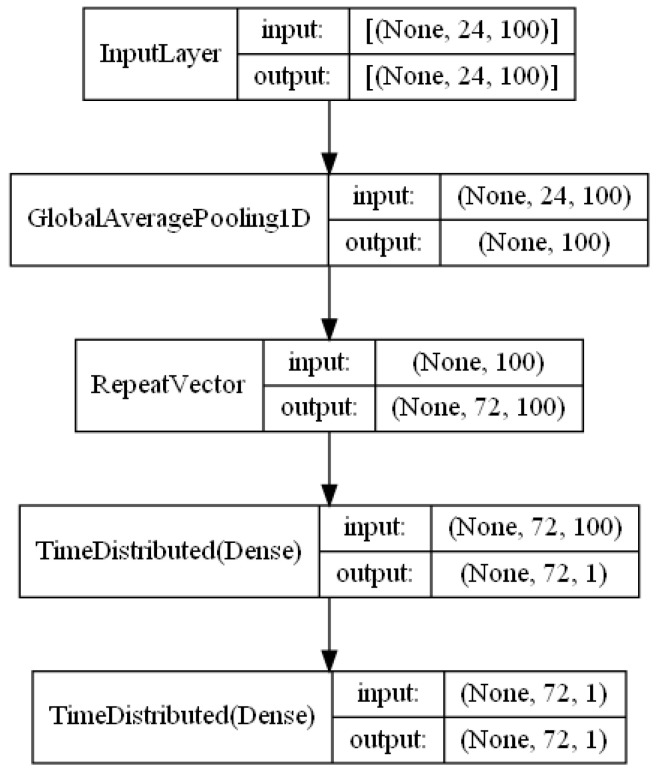
Functional unit that maps local sensor data to the target.

This model takes two inputs and there is a relatively independent functional unit that maps each input to the output (except at the last layer where the outputs are averaged during model training and execution). The functional unit for the left hand side is detailed in Figure 6, where there are four layers (if we do not count the Input layer). The GlobalAveragePooling1D layer calculates the average values for all samples in the lookback window for every input feature. Though the variance among samples over time are ignored due to this calculation, it helps to accelerate execution speed during model training and model prediction. The RepeatVector layer repeats its input (which is the output of its previous layer) a number of times which is equivalent to the size of the lookforward window. This operation creates identical but separate datasets which will be used to independently forecast values at different time steps in the lookforward window. Each copy of the dataset will be corresponding to one time step in the forecast window in this case. The TimeDistributed(Dense) layer builds multiple dense neural networks between all features in its input and all features in its output. The number of dense neural networks that are built depend on the number of time steps in the forecast window. In order to simplify the network architecture and improve efficiency, there are no interconnections among the different dense neural networks added on this layer. We have added another TimeDistributed(Dense) layer to the end and it helps to build a deep learning network. We can increase the number of output features from its previous layer to better utilize this model. In this particular case, we can remove the last TimeDistributed(Dense) layer as there is only one output feature per sensor.

**Figure 7:**
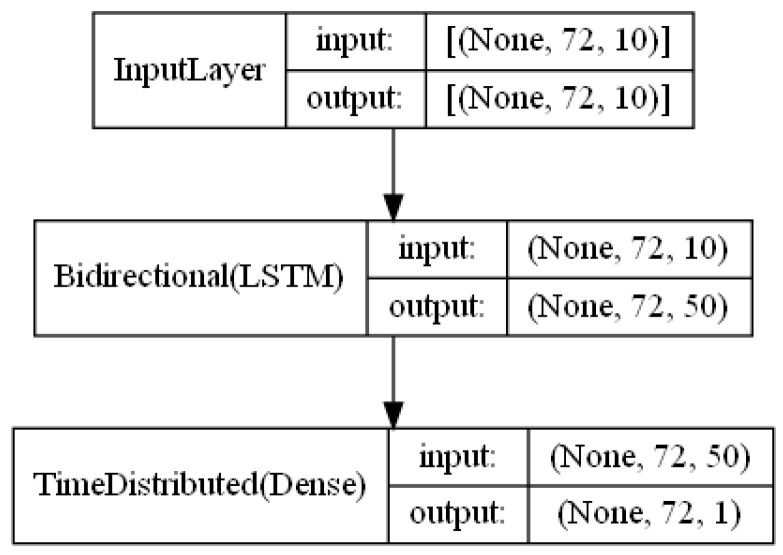
Functional unit that maps weather forecast to the target.

The functional unit for the right hand side is detailed in Figure 7. There are only two layers in this case (if we do not count the Input layer). The Bidirectional(LSTM) layer builds a bidirectional LSTM recurrent neural network that processes input information flow in both directions (i.e., future to past, and past to future). Without the bidirectional feature, an output value at time step 1 (acquired by returning the hidden states at different time steps) could only utilize input information from time step 1 (and therefore could have very strange values if the input value at time step 1 is strange), while an output value at time step n could utilize all input information from time step 1 until n. The using of bidirectional LSTM solves this problem by allowing all outputs to be dependent on all inputs. The output of Bidirectional(LSTM) layer has 50 features which is adjustable. Finally, we add a TimeDistributed(Dense) layer at the end to map the outputs from previous layer to the target output feature.

The current model architecture has been based on a set of models we have developed earlier [8], and has considered both performance and efficiency. For example, the using of the GlobalAveragePooling1D layer (which simply calculates the average of each feature over all timestamps in the lookback window) could greatly increase the execution speed without significantly decreasing the performance in comparison to the using of an LSTM layer or a bidirectional LSTM layer for the history input. In the graphs for the model architecture and for the functional units, we have ignored the regularization which have been implemented by using Keras L2 kernel regularizers and by randomly dropping neurons during training.

## 6. Results

In this Section, we present the test results from training the proposed machine learning model and also show the results of real-time forecast after the proposed method has been implemented in software and integrated into Kristianstad Municipality’s IoT portal (for real-time monitoring and decision-making).

### 6.1. Software Implementation

The software implementation is a time-consuming complex task. And the software that is used for model training and evaluation purpose is slightly different from the software that is used for real-time forecast. For evaluation purpose, only history data are used because we need to see the difference between the model prediction and the actual observation. Since we do not have history forecast data from SMHI, we have used local sensor data to estimate history forecast as shown in Figure 3. For real-time forecast, history data from a selected long period (e.g., three months) back in time is used for model training and model update (which happens every 24 h in our configuration). The most recent sensor data from a short period back in time (corresponding to the length of the lookback window if we do not consider that the calculation of those accumulated weather parameters in Figure 3 require data from a slightly longer period back in time) and the actual future weather forecast information from SMHI are used for model prediction. Algorithm 1 shows the pseudo code for model training and model prediction for real-world deployment.**Algorithm 1:** Pseudo code for model training and model prediction.
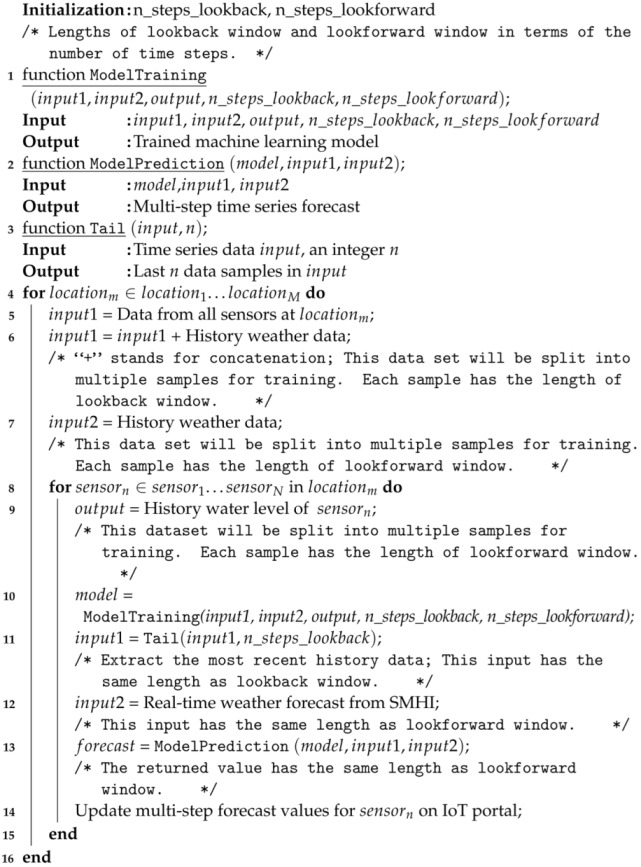


Though the proposed model has the ability to make forecast for a group of sensors at once, we found that the model performs best if it is custom trained and adapted for each individual target sensor. We basically implement this through two loops (demonstrated in Algorithm 1). In the outer loop, we go through each location (used for grouping sensors). Regardless which target sensor it is, the input data (used later for model training) come from all sensors belonging to the same location and also from the history global weather data (estimated using the information collected from all relevant sensors in the whole Kristianstad regardless of locations). In the inner loop, we pick a specific sensor in the selected location and train a model that is customized to this particular sensor. In this case, the output data is only associated with this particular sensor but the input data comes from all nearby sensors as explained earlier.

In our software product that has been deployed on the municipality’s server, we have skipped the outer loop as we have implemented a parallel processing which significantly improves the execution speed. In this case, data from IoT portal and from SMHI are firstly downloaded and pre-processed. We calculate global weather parameters from both IoT data and SMHI data as described in Section 3. The IoT data are divided into groups according to their physical locations. For each group, a separate process has been used for model training and prediction.

### 6.2. Model Evaluation

For performance evaluation purpose, we have used a history period of data (ranging from 27 October 2022 21:00:00 to 25 January 2023 21:00:00) for the selected location Degeberga (which is a village in the southern tip of Kristianstad). We have used different settings. The loopback window (n_steps_lookback) is set to the size of 24 h when the lookforward window (n_steps_lookforward) is set 24 h. The lookback window is set 72 h when the lookforward window is 72 h. This data set has been splitted into sub datasets for training (72%), validation (18%) and test (last 10%). In the following, we show the results of predicting water levels for two selected sensors (named respectively Sensor 1 and Sensor 2) in the village Degeberga.

**Figure 8:**
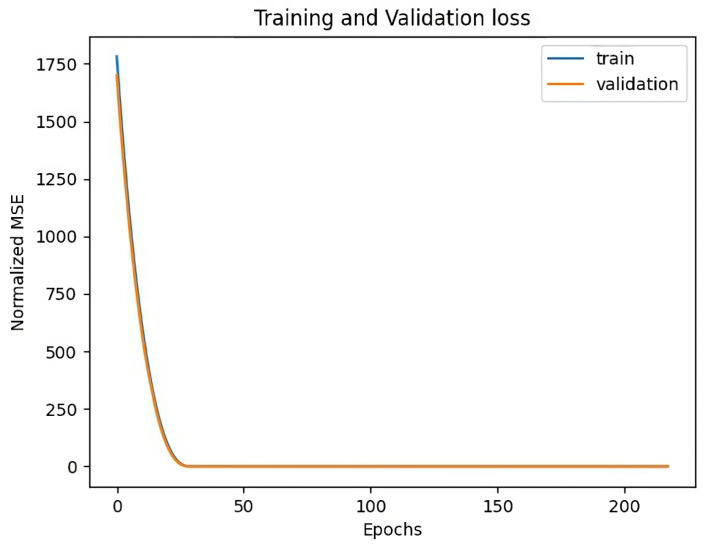
Training and Validation Losses for Sensor 1.

Figure 8 shows the training and validation losses for Sensor 1. At the end of model training, the training loss is as little as 0.20 while the validation loss is as little as 0.58. The losses has been measured using mean squared error (MSE) after data normalization. It can also been seen from the same figure that the training has been smooth and the model has converged after a few rounds. There is no over fitting of the model as the validation loss is so little.

**Figure 9:**
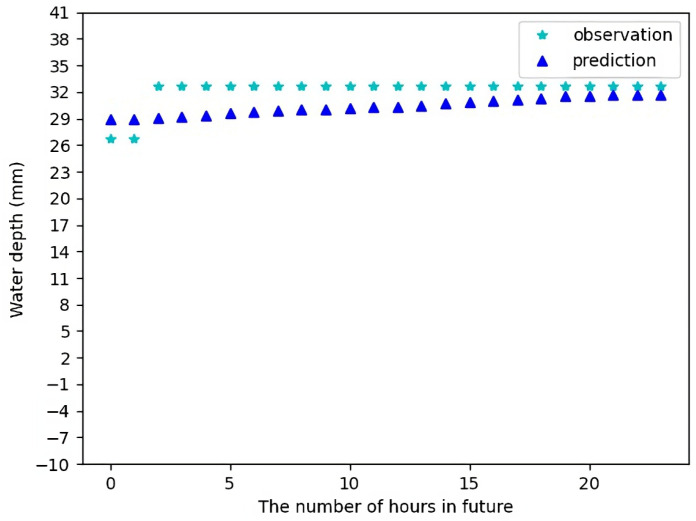
Water depth forecast for Sensor 1 for 24 h in future.

**Figure 10:**
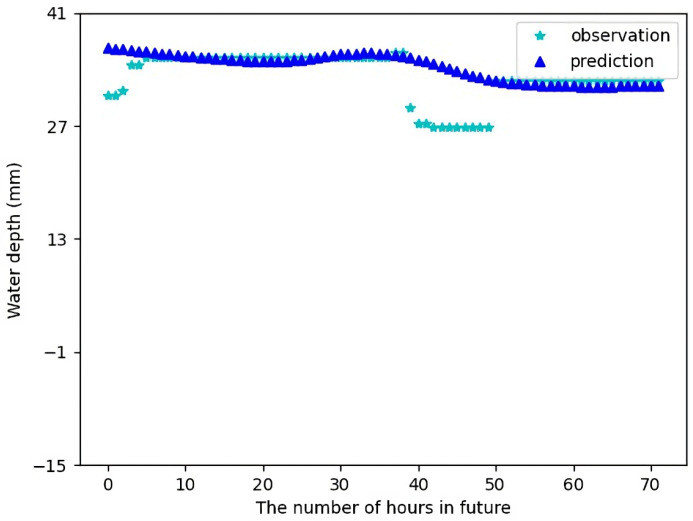
Water depth forecast for Sensor 1 for 72 h in future.

Figure 9 shows the forecast result for Sensor 1 for 24 h in future using the test data. Figure 10 shows the forecast result for Sensor 1 for 72 h in future using the test data. It can be seen there is a small difference between the forecast data and the actual observations. Most of the time, the forecast data makes quite accurate predictions, except in few cases when there are a sharp change in water levels which might be due to some random local conditions and have been treated as noise. In Figure 10, we can actually see that the actual observations follow the trend of our predicted values, which is quite good.

**Figure 11:**
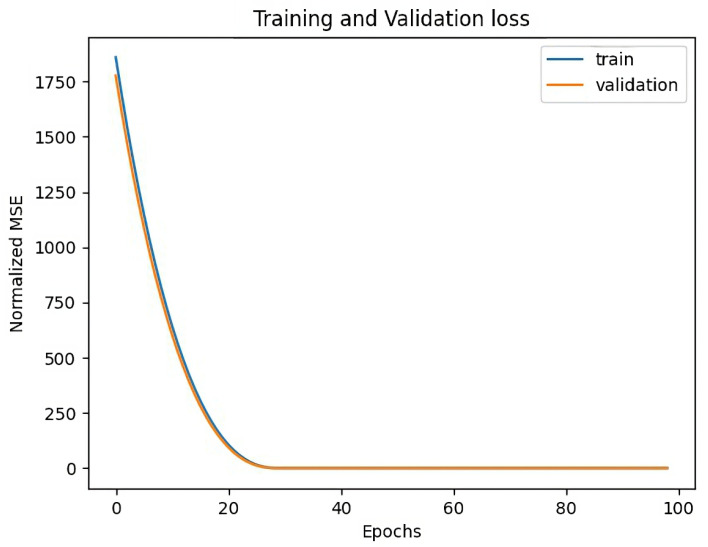
Training and Validation Losses for Sensor 2.

Figure 11 shows the training and validation losses for Sensor 2. At the end of model training, the training loss is as little as 0.24 while the validation loss finishes at 1.20 which is also quite small. The losses has been measured using mean squared error (MSE) after data normalization. For Sensor 2, we generally have the same good result as in the case of Sensor 1.

**Figure 12:**
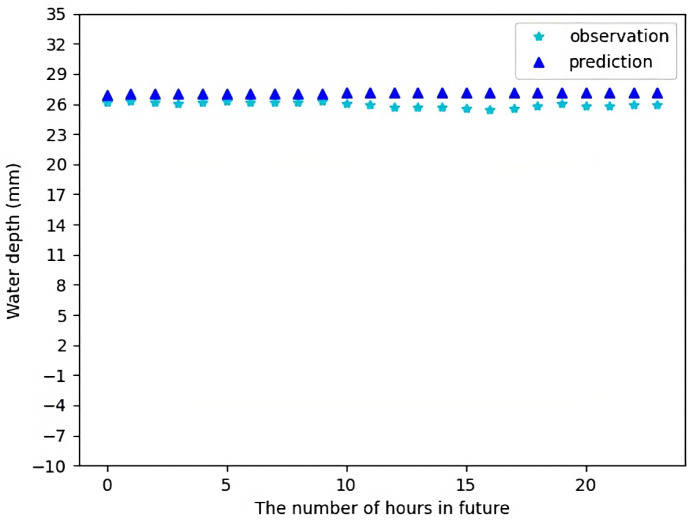
Water depth forecast for Sensor 2 for 24 h in future.

**Figure 13:**
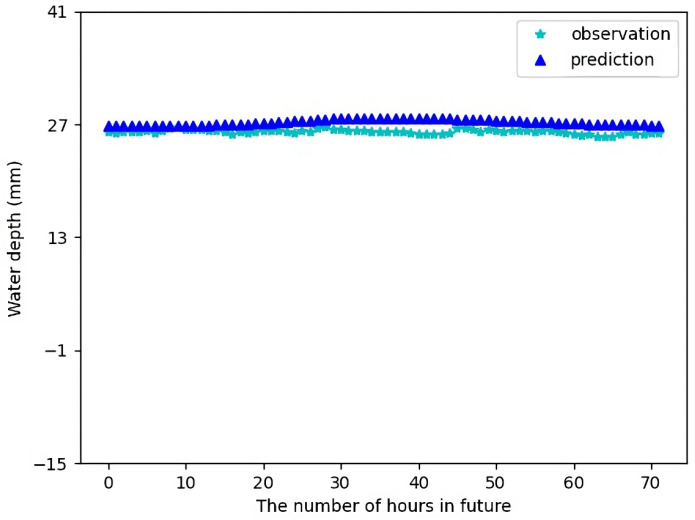
Water depth forecast for Sensor 2 for 72 h in future.

Figure 12 shows the forecast result for Sensor 2 for 24 h in future using the test data. Figure 13 shows the forecast result for Sensor 2 for 72 h in future using the test data. In this case, the differences between the forecast data and the actual observations are also quite small as in the case of Sensor 1.

### 6.3. Forecast at Real Time

After deployment, the machine learning model is (re)trained every 24 h for each individual target sensor. Using the real time weather forecast data for the next 10 days from SMHI, the model can make water level predictions for up to 10 days in the future (with forecast resolution decreased after 72 h due to low resolution data from SMHI). The results of future forecast values are organized in JSON format (with timestamps and values) and reported to the municipality’s IoT portal server via HTTP request.

**Figure 14:**
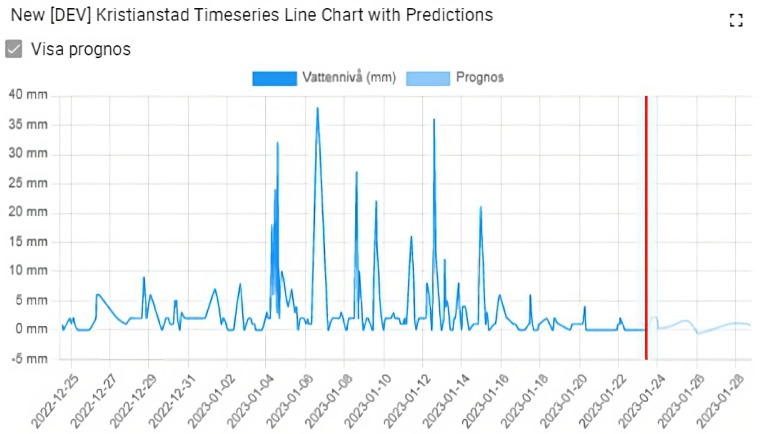
Real-time forcast data shown on IoT portal. Permission to publish is acquired from Kristianstad Municipality.

Figure 14 shows an example of the real-time forecast data shown on IoT portal in the same graph with history observations. The red line in the figure separates the future and the past. The forecast data is of very high quality.

## 7. Discussion

In this article, we have presented very good data forecast results for water levels in Kristianstad. We have largely adopted a divide-and-conquer approach in solving the forecast problem. Firstly, it was proven that the number of dimensions is too high if we involve all parameters from all sensors in the model. We then propose to divide the sensors and group them according to their physical locations. This has led to trainable models with reasonably good results. We then further propose to customize the model to each particular sensor. In comparison to a group training, the customized model has led to even better results as shown in this article. A tradeoff in this process could be between the number of models you need to train and the performance in terms of forecast accuracy.

During the development time, many sensors from one of the sensor providers has shown to be problematic with error readings. This problem has not been fully solved yet at the time of writing. This potentially has affected model training and forecast accuracy. As we are using a small lookback window (24 h or 72 h), the time-varying errors possibly do not have a large effect on the forecast (if we still make comparison to the actual readings from the same sensor). But if we are going to train the model using a larger lookback window, the time-varying error patterns will be able to confuse the model during training and even lead to non-stable model parameters. It is however desired to have larger lookback window if we aim to catch long-term trend such as the seasonal effects.

In addition to rain, landscape and the topology of water pipe network, the water levels are also affected by pumping which is currently manually controlled. Once a pump is started, water levels on the two sides of the pump can be dramatically changed during a short time. In comparison to the slow changing water levels without pump effects, this is a totally different phenomenon and should be better explained by separate models. The current model has not looked into depth on pump effects and has learned the relations blindly. This can be something that can be improved in the future.

The history data of SMHI weather forecast is not available for the model development. Though we believe the current proposed approach of using local sensor readings to estimate history data of SMHI is a good method, it is still interesting to make some comparisons if we can train a model using real SMHI history forecast data. It depends on what quality of data SMHI has for its forecast, the estimation using local sensors can be a better approach as it represents the ground truth of the actual observed local weather.

## 8. Conclusions and Future Works

We have presented and implemented a machine-learning model on predicting future water levels in the city Kristianstad in Sweden. This model has integrated inputs from local sensors and data from 3rd party weather forecast service. A divide-and-conquer approach (by grouping sensors) has been adopted to treat the high-dimensional data inputs from over 550 sensors (and this number has been increased to 650 at the end of the project). The proposed method has been shown to be able to predict future water levels with high accuracy. The software implementation has been successfully deployed and integrated in the real-time water monitoring system of Kristianstad. The forecast system will be able to forecast flood threats multiple days in advance and will facilitate early decision-making (such as decisions on starting pumps and on evacuation).

There can be many interesting future developments. One future development will be to continue improving the forecast accuracy by looking at sensor data from a long history back in time. We do not have that amount of data yet as the sensors are only recently deployed. We can also try to optimize the grouping of sensors. We have grouped sensors according to their physical locations but the number of sensors per group is not even. We can use smaller groups for those densely deployed regions and a smaller group potentially contribute to better results if we think about the achieved dimension reduction from a smaller amount of input parameters.

Another interesting application will be look at anomaly data in water wells. This will help to detect water leakage due to inflow and infiltration. Eventually this will help underground water pipe maintenance and improve the capacity of the wastewater treatment plant by reducing inflow and infiltration from ground water. This article has used local IoT data to estimate history weather forecast. This opens window for a new interesting application by integrating local IoT data (which are distributed in different cities) with the national and international weather forecast systems.

## Figures and Tables

**Table 1:** Parameter values used in our model description (only for illustration purpose). These parameter values can be different from the ones used in Section 6.

Parameter	Value	Definition
n_samples	None	The number of training samples which is not specified in the model.
n_input_features	100	The number of input features including all features from all local sensors. But irrelevant features and dependent features are removed. The history data can also include history weather forecast.
n_smhi_features	10	The number of features in the SMHI weather forecast data.
n_output_features	1	The number of output features which is only water depth in this case. It is possible to predict this feature for multiple sensor locations at once and then we have n_output_features ≥ 1.
n_steps_lookback	24	The number of history sensor observations that will be used in a training or prediction sample. It is a 24-h window in this case.
n_steps_lookforward	72	The number of future observations that will be predicted. It is a 72-h window in this case.

## Data Availability

Weather forecast data from SMHI is avaiable on SMHI website [23]. Other data are not available for public.

## Abbreviations

The following abbreviations are used in this manuscript:

SMHI	Swedish Meteorological and Hydrological Institute
LSTM	Long short-term memory neural network
HTTP	Hypertext Transfer Protocol
JSON	JavaScript Object Notation
ANN	Artificial neural network
MSE	Mean squared error
IoT	Internet of Things
AI	Artificial Intelligence
